# RLT-S: A Web System for Record Linkage

**DOI:** 10.1371/journal.pone.0124449

**Published:** 2015-05-05

**Authors:** Abdullah-Al Mamun, Robert Aseltine, Sanguthevar Rajasekaran

**Affiliations:** 1 Department of Computer Science and Engineering, University of Connecticut, Storrs, Connecticut, United States of America; 2 Institute for Public Health Research, University of Connecticut, East Hartford, Connecticut, United States of America; Leibniz Institute for Age Research, GERMANY

## Abstract

**Background:**

Record linkage integrates records across multiple related data sources identifying duplicates and accounting for possible errors. Real life applications require efficient algorithms to merge these voluminous data sources to find out all records belonging to same individuals. Our recently devised highly efficient record linkage algorithms provide best-known solutions to this challenging problem.

**Method:**

We have developed RLT-S, a freely available web tool, which implements our single linkage clustering algorithm for record linkage. This tool requires input data sets and a small set of configuration settings about these files to work efficiently. RLT-S employs exact match clustering, blocking on a specified attribute and single linkage based hierarchical clustering among these blocks.

**Results:**

RLT-S is an implementation package of our sequential record linkage algorithm. It outperforms previous best-known implementations by a large margin. The tool is at least two times faster for any dataset than the previous best-known tools.

**Conclusions:**

RLT-S tool implements our record linkage algorithm that outperforms previous best-known algorithms in this area. This website also contains necessary information such as instructions, submission history, feedback, publications and some other sections to facilitate the usage of the tool.

**Availability:**

RLT-S is integrated into http://www.rlatools.com, which is currently serving this tool only. The tool is freely available and can be used without login. All data files used in this paper have been stored in https://github.com/abdullah009/DataRLATools. For copies of the relevant programs please see https://github.com/abdullah009/RLATools.

## Introduction

Record linkage has evolved as a crucial problem in many areas of science and engineering. A large number of health agencies store medical records of patients [1, 2]. Finding data of an individual across these sources requires efficient algorithms. Record linkage has also applications in disease evolution [3, 4], master data management, copy detection in digital documents [5, 6], historical data management, and so on.

Record linkage collects records of same individuals from multiple data sources possibly having some corrupted records due to typo, phonetic similarity, etc. Now-a-days data of an individual reside across multiple databases and at the same time data agencies keep records of millions of people. Accuracy as well as time efficiency in finding all the records of an individual make the problem challenging. A naive algorithm compares each pair of records and measures similarities. This method is very time consuming. Many algorithms have been devised to improve this naive algorithm [7–11]. We have already proposed efficient and effective sequential and parallel record linkage algorithms [12], which outperform previous best-known record linkage algorithms [13]. Our methods use single linkage hierarchical clustering which generates a dendrogram. By applying a threshold value on this dendrogram we get our expected clusters for individuals.

A large number of record linkage tools are widely available. Java-based fine-grained probabilistic record integration and linkage tool (FRIL) is an open source tool, which has support for parameters configuration and can handle millions of records [14, 15]. Another widely used record linkage tool is FEBRL (Freely Extensible Biomedical Record Linkage) which performs data standardization as well as probabilistic record linkage of one or more files [16].

In this paper we present details on our record linkage tool, RLT-S, which implements the record linkage algorithm based on single linkage clustering of [12]. This tool is freely available in www.rlatools.com website. The website also provides proper instructions, submission history and some other necessary features to ease the usage of the tool. The tool generates a well-formatted output to facilitate user perception. In this paper we describe the functionalities of the tool as well as necessary parameters for input handling, linkage processing, and generation of output.

## Implementation

RLT-S is a Java implementation of sequential RLA (Record Linkage Algorithm) [12]. This algorithm clusters records of individuals using single linkage hierarchical clustering. It merges records from all the data sets as if they were from one data set. Therefore the performance of the algorithm is independent of the number of input data sets. It sorts records on common attribute fields using radix sort. Sorting helps us to separate duplicate records, which indicates exact matching. Real life applications do not contain much error. Therefore the sorting phase reduces the size of the unified data set into a smaller data set with no exact duplicates of records. Next phases work on only the representative records from all the exact match clusters. We call the first record of each cluster as the representative record of that cluster. Our RLA employs single linkage hierarchical clustering. Comparison between each pair of records consumes a lot of time. To facilitate finding groups of similar records it employs blocking on a specified attribute field. It finds linkages among those records in a block. We use l-mers (i.e., substrings of length l) of the attribute field for blocking. Any L-length record will be present in (L—l + 1) blocks. In this way different blocks are connected. We then employ hierarchical clustering with single linkage by measuring distances between pairs of records in a block using any combination of edit distance, reversal distance, and truncation distance methods. If we consider each record as a vertex in a graph and linkage as an edge between two vertices, then we get our desired graph. We remove multiple edges and self-loops from this graph. Each connected component of this graph is a cluster of records of an individual. The algorithm outputs these clusters and all their identical records generated by exact matching as final clusters.

The website and the associated tool perform record linkage among one or more data files. We have simplified the usage of the tool by minimizing queries and text input. Whenever possible we have provided drop-down lists to select possible values. Generated output is also well formatted so that the users can easily identify records of an individual.

## Results and Discussion

**Fig 1:**
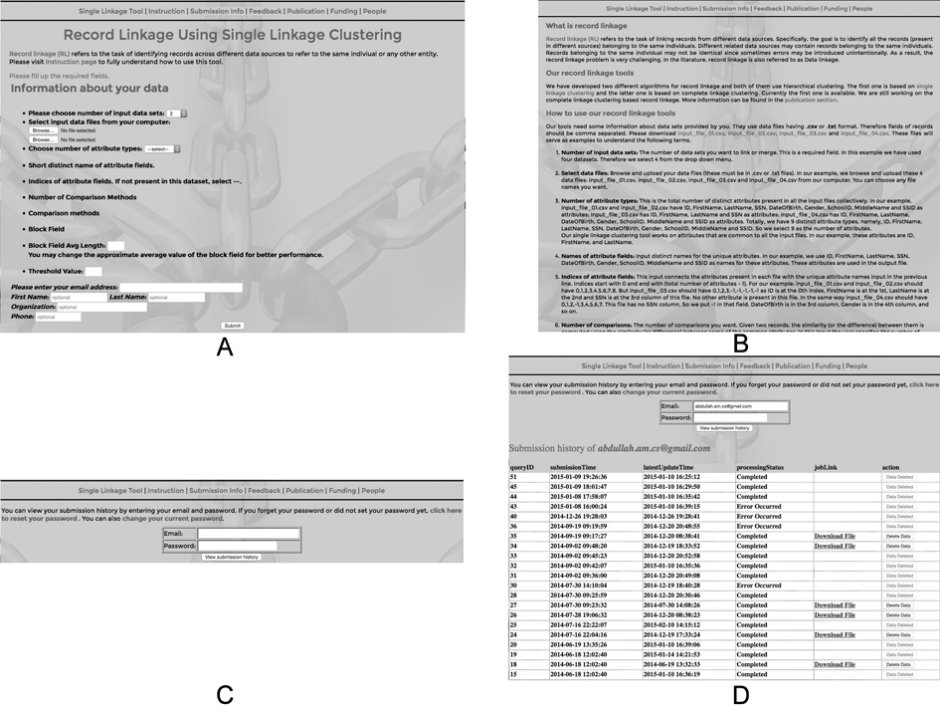
Web-based user interface. (A) shows the first and main page of the website, where users select data files, choose configurations and submit them. (B) is the instruction page. Users can view their submission history through login (C). (D) shows a sample submission history page.

http://www.rlatools.com hosts RLT-S tool and provides all the other necessary features to ensure the best functioning of the tool. Anyone can use this freely available tool with or without login information. Registered users have the facility to view all of their submissions information and outputs. But without login also users can use the tool and find their outputs using links via email. Currently we keep all the output files in our server so that the users can view and download them at any time. Fig 1 shows a diagram of the website pipeline.

Our tool requires some specific information. Some of these are required, some are highly recommended and a few of them are optional. We have set default values for some attributes if the users do not want to input them. RLT-S works in three separate phases. The first and the third phases work with the input and the output. The second phase tunes parameters and controls the working process of the tool.

### Input data sets and configurations

The tool starts with reading and organizing the input data sets. The number of input data sets is required to browse those data files from the user’s computers. It accepts. txt or. csv extension files where the attributes of each record are comma separated and each record is in a separate line. We illustrate the working of RLT-S with an example. This example pertains to Tables 1, 2, and 3.

**Table 1:** Records for 5 people having 9 attributes.

ID	FN	LN	SSN	DoB	G	SchID	MN	SSID
1	Risa	Pierce	133183594	09261990	M	1524	Vesta	0676221410
2	Maile	Kramer	135370878	07261991	F	1526	Lenna	0957261480
3	Kimberly	Battle	141274186	04071982	F	1527	Jacki	0144591609
4	Kamal	Mcclain	148965694	10091991	M	70000	Luisa	0278635088
5	Yvonne	Vaughan	153614228	02061992	F	70003	Basil	0368901550

Each row of the table represents each row of Input01.csv file.

**Table 2:** Records for 5 people having 4 attributes.

ID	First Initial	Last Name	Social Security Number
1	R	Pierce	133183594
2	M	Kramer	135370878
3	K	Battle	141274186
4	K	Mcclain	148965694
8	L	MUELLER	184498846

Each row of the table represents each row of Input02.csv file.

**Table 3:** Records for 5 people having 8 attributes.

ID	FirstName	LastName	DateOfBirth	Gender	SchID	MN	SSID
1	RISA	PIERCE	09261990	M	001524	VESTA	0676221410
2	MAILE	KRAMER	07261991	F	001526	LENNA	0957261480
3	KIMBERLY	BATTLE	04071982	F	001527	JACKI	0144591609
5	YVONNE	VAUGHAN	02061992	F	070003	BASIL	0368901550
8	KELSIE	MUELLER	01131992	M	070020	JAKE	7243583370

Each row of the table represents each row of Input03.csv file.

Table 1 shows records of five people. Each record has ID, FN, LN, SSN, DoB, Gender, SchID, MN, and SSID. Table 2 has also 5 records having ID, First Initial, Last Name and Social Security Number as attributes. We have another 5 records from Table 3, each of which has ID, FirstName, LastName, DateOfBirth, Gender, SchID, MN, and SSID as attributes. These 3 tables have different numbers of attributes. We see that the attribute names are quite different from each other although some of them represent the same type. FN, First Initial and FirstName represent first name of a person. Similarly LN, Last name and LastName are similar types.

**Fig 2:**
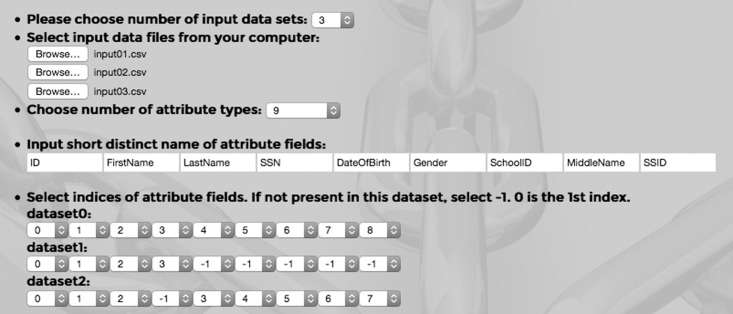
Screenshot of input parameter selection for our 3 example files.

Consider the task of integrating these 3 tables using our tool. The first required field in RLT-S is the number of input data sets. In this case we select 3 from drop-down list. Then we have to select input files from our computer. As we see some attribute type names are different although they represent the same attribute, we remove first row from each input file. There are 3 browse fields. We browse our computer and select one file at a time. Input01.csv is added at the first browse field, Input02.csv and Input03.csv are added at the second and third fields, respectively. Next required field is the number of attribute types. In this example we see that 9 unique attribute types are present. So we select 9 from the drop-down list. We have seen that the same type has been represented differently in these files. So we choose 9 suitable names for these attribute types, for example, ID, FirstName, LastName, SSN, DateOfBirth, Gender, SchoolID, MiddleName and SSID. Now we have to connect these names with attribute field names of each data set. We note that ID is the 0^th^ index of 1^st^ input file, FirstName is the 1^st^, LastName, SSN, DateOfBirth, Gender, SchoolID, MiddleName and SSID are subsequent indices. So we select 0, 1, 2, 3, 4, 5, 6, 7, 8 from the drop-down list for dataset0 or Input01.csv. For dataset1 or Input02.csv, ID is the 0^th^, FirstName is the 1^st^, LastName is the 2^nd^ and SSN is the 3^rd^ index. This file has no DateOfBirth, Gender, SchoolID, MiddleName and SSID attribute fields. So we select -1 for each of them. Dataset1 should have 0, 1, 2, 3, -1, -1, -1, -1, -1. Input03.csv has no SSN field. So we put -1 for this index. DateOfBirth is at index 3 of this file, Gender is at 4, and so on. Therefore we select 0, 1, 2, -1, 3, 4, 5, 6, 7 for this dataset. This is the last step of the input phase. Fig 2 shows the above selection of input files.

### Linkage parameters

**Fig 3:**
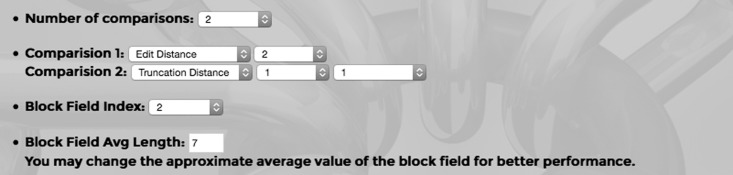
Screenshot of linkage criteria for our 3 example files.

This stage handles the working process of the tool. RLT-S works on common attributes across all the data sets. We need a measure of distance between two attributes (i.e., two strings of characters). RLT-S supports three distance measures. The tool has three types of fields for each comparison. The first field is to select the comparison method (i.e., the distance measure), second one is for selection of the index on which the selected comparison method should be applied and the third type is truncation count, which is the number of characters of the selected attribute that should be used for comparison. We integrate three different comparison methods. The first comparison method is the edit distance calculation. Edit distance or Levenshtein distance measures the minimum number of edit operations required to transform one string to another. Operations include insertion, deletion and substitution. For example, consider the strings A = "computer" and B = "conuterr". If we substitute 'n' to 'm', insert 'p' after this 'm' and delete the last 'r' from B, we get A. Edit distance method needs at least three operations to transform B to A. To use this method, the user has to select an index of the common attribute on which this method will apply. Another distance measure is the reversal distance. Consider a string of two attributes separated by comma, A = "James,Hudson" and another string B = "Hudson,Hames". This method first calculates the edit distance between A and B. We note that a large number of operations are required to transform one string to another. Then it alters the positions of the two strings of B generating B' = "Hames,Hudson" and then measures the distance between A and B'. It finds that only one operation is needed for the transformation. The reversal distance measure is defined as the minimum of the distance between A and B and the distance between A and B’. This method is very useful for the first name and the last name attributes or any other related attributes as users may occasionally input the first name in the last name field and vice versa. Reversal edit distance method needs two attributes to work with. So there are two index fields, each of which should be a unique common attribute index. Truncation distance method is the last method used in our tool. This method is the same as the edit distance method except that it only compares truncation count number of initial characters of both attributes. For example, if A = "James" and B = "J" and if the truncation count is 1, this method calculates the edit distance between A' = "J" and B' = "J". Truncation often occurs for first names as some sources keep only the first name initials. Our single linkage-clustering algorithm reduces a major portion of time for linkage calculation by using blocks on a specific attribute. Each block stores information of similar records. Therefore output accuracy also depends on the choice of the attribute field and its average length. In [12], we have used the last name as the blocking field because the last name was the most appropriate attribute in our collected records. Any other important and reliable common attribute may be the blocking field as well. In most of the cases there is no way to measure a perfect average length of the block field. But an approximate average length makes our job easy. If the user does not fill in this field, the tool uses 7 as the default value. In [12], we show how the value of k affects the blocking performance. In our example we have used 2 comparisons, edit distance calculation on the last name, and truncation distance method on the first name with a truncation count of 1. Our linkage criteria are shown in Fig 3.

### Output

The third stage requires some information to generate and send outputs. RLT-S employs hierarchical clustering and generates a dendrogram. A dendrogram represents clusters produced by hierarchical clustering in a tree in a well-organized way. A threshold value is needed to output the desired clusters. This threshold value says how many errors RLT-S endures. To understand fully the threshold value, let us consider another example. One record has James, Rodriguez, and 01011990 for FirstName, LastName and DateOfBirth fields, respectively, and another record has Rodriuez, 123456789, and Hames as LastName, SocialSecurityNumber and FirstName attributes. We see that 'James' has been transformed into 'Hames' which indicates that one substitution is needed to correct it and one character has been deleted from 'Rodriguez'. The matching process finds 2 errors between these two records. If we input a threshold value of 1, then the tool produces 2 clusters, each of which contains only one record. But if the threshold value is at least 2, only one cluster having these two records will be generated. A threshold value of 0 generates exact clustering in which every record matches on common attributes. If the users do not input any value, the tool uses the most used threshold value of 1 as the default value. User's email address is required as the output link is sent to this address. For our example of 3 input files, if we choose 1 as the threshold value, then RLT-S will generate the output shown in Table 4.

**Table 4:** Generated output for our example data sets.

Cluster ID	File Name	ID	First Name	Last Name	SSN	Date Of Birth	Gender	School ID	Middle Name	SSID
1	Input02.csv	1	r	pierce	133183594					
1	Input01.csv	1	risa	pierce	133183594	09261990	m	1524	vesta	0676221410
1	Input03.csv	1	risa	pierce		09261990	m	1524	vesta	0676221410
2	Input02.csv	2	m	kramer	135370878					
2	Input01.csv	2	maile	kramer	135370878	07261991	f	1526	lenna	0957261480
2	Input03.csv	2	maile	kramer		07261991	f	1526	lenna	0957261480
3	Input02.csv	3	k	battle	141274186					
3	Input01.csv	3	kimberly	battle	141274186	04071982	f	1527	jacki	0144591609
3	Input03.csv	3	kimberly	battle		04071982	f	1527	jacki	0144591609
4	Input02.csv	4	k	mcclain	148965694					
4	Input01.csv	4	kamal	mcclain	148965694	10091991	m	70000	luisa	0278635088
5	Input01.csv	5	yvonne	vaughan	153614228	02061992	f	70003	basil	0368901550
5	Input03.csv	5	yvonne	vaughan		02061992	f	70003	basil	0368901550
6	Input03.csv	8	kelsie	mueller		01131992	m	70020	jake	7243583370
6	Input02.csv	8	l	mueller	184498846					

### Submissions history

Users may want to check their previous submissions and outputs. The website allows users to login to view their submissions history. They can check query id, submission time, processed time, download link, etc. of all the submitted jobs.

Any user can use the tool without login information. Valid email address is needed to get the download link of the output. First time users need to reset their password from the “submission info” section to be registered. They can also change their current password from this page.

### Feedback

The website provides a feedback option for further improvements to RLT-S. Users can select a feedback type and post comments. We will study the feedbacks very carefully and modify the system accordingly.

RLT-S application has been implemented in Java. We have used Apache server with MySQL to host the website. A service works in the background to trigger the application when a new job is posted. This service also keeps track of the finished jobs, updates database and sends email to corresponding users. MySQL database stores users' information and their submission history. Our tool takes negligible amount of time for thousands of records. If needed, we will integrate our parallel implementation of single linkage clustering algorithm into this website in future.

## Comparisons

FEBRL and FRIL are well known and widely used freely available record linkage tools. These tools perform standardization or deduplication of a file or linkage between two files. On the other hand, our system RLT-S can handle any number of input datasets. Several experimental results reported in [12] show that our RLA algorithm outperforms previous best-known algorithms for error-induced datasets. Those experiments also describe the process of choosing suitable threshold values for different datasets. Our algorithm achieves around 98% accuracy on four real datasets having 1 million records in total. This algorithm was 70 times faster than the previous best-known algorithm, TPA (FCED) [13], for these datasets.

Many of the available record linkage tools achieve very good accuracy, but they suffer from higher time complexities to generate linkages among datasets. Efficient TPA (FCED) (Two Phase Algorithm with Faster Computation of the Edit Distance) has been compared with FEBRL for two datasets of different sizes [13]. In this paper we go through some experiments which use the same parameter configurations as [13]. All of these four tools, namely RLT-S, FEBRL, FRIL, and TPA (FCED), achieve 100% accuracy for these simulated datasets. Table 5 shows computation times of these four tools for 1000, 2000, 3000, 4000, and 5000 records. In this table the notation (X, Y) stands for the number (X) of records in the first file and the number (Y) of records in second file.

**Table 5:** Time comparison of RLT-S with FEBRL, FRIL, and TPA (FCED).

Tool Name	(1000, 1000)	(2000, 2000)	(3000, 3000)	(4000, 4000)	(5000, 5000)
RLT-S	95	110	142	212	237
FEBRL	330	834	1630	2770	4150
FRIL	841	1992	3555	6043	8683
TPA (FCED)	172	223	274	360	433

Times shown are in milliseconds. Computation times are taken for (number of records in first file, number of records in second file).

We see that FEBRL is performing better than FRIL for each of these data sets. TPA (FCED) is faster than FEBRL that is also shown in [13]. RLT-S outperforms all of these tools. Our tool performs its best for real datasets, where the possibility of error occurrences and the number of errors in the input datasets are low.

## Conclusions

We have developed a record linkage tool called RLT-S. This tool is integrated into www.rlatools.com. This site contains instructions for usage as well as submissions history and some other useful features. RLT-S is the implementation of our efficient sequential record linkage algorithm, which has outperformed previous best-known algorithms in this area [12]. The tool requires very compact but necessary parameter selections for expected output in the shortest possible time. We also track user movements through this website. Tracking information and user feedback will help us to fine-tune the features and functionalities of RLT-S.

## References

[1] Clark DE (2004) Practical introduction to record linkage for injury research. Injury Prevention 10(3): 186–191. 15178677 10.1136/ip.2003.004580 PMC1730090

[2] Victor TW, Mera RM (2001) Record linkage of health care insurance claims. Journal of the American Medical Informatics Association 8(3): 281–288. 11320072 10.1136/jamia.2001.0080281 PMC131035

[3] Fayyad U, Piatetsky-shapiro G, Smyth P (1996) From data mining to knowledge discovery in databases. AI Magazine 17(3): 37.

[4] Maizlish NA, Herrera L (2005) A record linkage protocol for a diabetes registry at ethnically diverse community health centers. Journal of the American Medical Informatics Association 12(3): 331–337. 15684130 10.1197/jamia.M1696 PMC1090465

[5] Brin S, Davis J, Garcia-Molina H (1995) Copy detection mechanisms for digital documents. ACM SIGMOD Record 24(2): 398–409.

[6] Shivakumar N, Garcia-Molina H (1996) Building a scalable and accurate copy detection mechanism. Proceedings of the First ACM International Conference on Digital Libraries. ACM: 160–168.

[7] Gomatam S, Carter R, Ariet M, Mitchell G (2002) An empirical comparison of record linkage procedures. Statistics in Medicine 21(10): 1485–1496. 12185898 10.1002/sim.1147

[8] Gu L, Baxter R, Vickers D, Rainsford C (2003) Record linkage: Current practice and future directions. CSIRO Mathematical and Information Sciences Technical Report 3: 83.

[9] Zhao Y, Karypis G (2002) Evaluation of hierarchical clustering algorithms for document datasets. Proceedings of the Eleventh International Conference on Information and knowledge management. ACM: 515–524.

[10] Rajasekaran S (2005) Efficient parallel hierarchical clustering algorithms. IEEE Trans. Parallel Distrib. Syst. 16(6): 497–502.

[11] Mi T, Aseltine R, Rajasekaran S (2008) Data integration on multiple data sets. Proceedings of the 2008 IEEE International Conference on Bioinformatics and Biomedicine: 443–446.

[12] Mamun A-A, Mi T, Aseltine R, Rajasekaran S (2014) Efficient sequential and parallel algorithms for record linkage. Journal of the American Medical Informatics Association: JAMIA 21(2): 252–262. doi: 10.1136/amiajnl-2013-002034 24154837 10.1136/amiajnl-2013-002034 PMC3932463

[13] Mi T, Rajasekaran S, Aseltine R (2012) Efficient algorithms for fast integration on large data sets from multiple sources. BMC Medical Informatics and Decision Making 12(1): 59. 22741525 10.1186/1472-6947-12-59 PMC3439324

[14] Jurczyk P, Lu JJ, Xiong L, Cragan JD, Correa A (2008) Fine-grained record integration and linkage tool. Birth Defects Research Part A: Clinical and Molecular Teratology 82: 822–829. doi: 10.1002/bdra.20521 18985680 10.1002/bdra.20521

[15] Jurczyk P, Lu JJ, Xiong L, Cragan JD, Correa A (2008) FRIL: A tool for comparative record linkage. AMIA Annual Symposium Proceedings: 440–444. 18998844 PMC2656092

[16] Christen P (2008) Febrl: a freely available record linkage system with a graphical user interface. Proceedings of the Second Australasian Workshop on Health Data and Knowledge Management 80: 17–25.

